# Editorial: Personality and Disease: New Directions in Modern Research

**DOI:** 10.3389/fpsyg.2021.828337

**Published:** 2022-02-16

**Authors:** Federica Galli, Annalisa Tanzilli, Maurizio Pompili, Michael Bagby

**Affiliations:** ^1^Department of Dynamic and Clinical Psychology, and Health Studies, Faculty of Medicine and Psychology, Sapienza University of Rome, Rome, Italy; ^2^Department of Neurosciences, Suicide Prevention Center, Sant'Andrea Hospital, University of Rome, Rome, Italy; ^3^Department of Psychiatry, University of Toronto, Toronto, ON, Canada

**Keywords:** personality, disease, chronic pain, cardiovascular disease, psychopathology

## Introduction

For decades, research focused on chronic illness and psychological issues has evidenced a pervasive association between psychopathological conditions (mostly, anxiety, and depression) and different diseases. These comorbidities indicate that the connections between symptom presentations and physical pathologies do not happen by chance, but they reflect causal and salient mechanisms that are still unknown. We think personality may play a crucial role in better understanding the body-mind interplay.

A recent and ever-growing corpus of evidence has shown that both specific personality traits (especially, the dimensions of Neuroticism and [low] Conscientiousness included in the Five-Factor Model; for an overview see Korotkov and Hannah, 2004; Dammeyer and Zettler, 2018) and personality disorders—defined as enduring maladaptive patterns of behavior, cognition, and inner experience—co-occur with various medical conditions, such as cardiovascular diseases, cancer, chronic pain, headaches, psoriasis, and other skin disorder, etc. (e.g., Rubino et al., 1995; Galli et al., 2011, 2017, 2019a,b; Levine et al., 2021). Moreover, research has indicated that dysfunctional personality patterns negatively affect the clinical prognosis, increasing the risk of poor health outcomes (e.g., Johansen, 2018).

In the light of the clinical and empirical literature in this field of investigation, it is clear the need for exploring the complex mechanisms and pathways that potentially underlie the relationship personality/illness to accumulate knowledge, promote more accurate diagnoses, and inform effective interventions that are tailored on specific patients' characteristics.

The present Special Issue comprises six highly multidisciplinary papers, covering different clinical and research areas on the associations between personality and diseases. Notably, three contributions (Cavicchioli et al., 2021; Durosini et al., 2021; Galli et al., 2021) have provided a theoretical overview of current knowledge derived from the scientific literature on the role of personality traits or syndromes in developing and maintaining various medical conditions (especially, oncological diseases, cardiovascular pathologies, overweight and obesity, chronic pain presentations). All the other contributions (Buratta et al., 2021; Lanzara et al., 2020; Mokhtar et al., 2020) have described and addressed the findings of empirical investigations on this specific Research Topic, discussing clinically meaningful implications and suggesting best practices for achieving successful intervention outcomes.

Looking in more detail all the Special Issue's contents, Cavicchioli et al. focused on the mutual relationships between maladaptive emotion regulation strategies and physical diseases in patients presenting with Borderline Personality Disorder (BPD). Consistent with a vast corpus of empirical evidence, the authors documented strong functional associations between meaningful vulnerabilities with emotion regulation and medical pathologies (e.g., cardiovascular and metabolic diseases, obesity-related pathologies, chronic pain) in borderline populations. Noteworthy, they proposed Dialectical Behavior Therapy (DBT; Linehan, 1993) as a well-validated and helpful therapeutic approach that could address pervasive and dysfunctional emotion regulation processes in BPD patients, reducing psychical diseases and promoting their well-being and psychological health.

The systematic review by Galli et al. provided a comprehensive picture of studies exploring the impact of personality on the onset of colorectal cancer (CRC). The authors identified the harmful influence of emotional dysregulation processes (mainly involving anger and hostility), and the potentially protective role of specific modes of relatedness with others (e.g., self-centeredness and independence described as self-defensive and self-interest-oriented attitudes). Although these findings are very thoughtful, the limited number of studies and the heterogeneity of the tools and methodologies employed in these investigations, did not allow to draw general and comprehensive conclusions. Future research is needed to shed light on the complex connections between personality dimensions and colorectal cancer.

Durosini et al. summarized and analyzed the current scientific evidence about the associations between personality traits and cardiotoxicity in anticancer treatments. The authors highlighted the crucial role of personality in developing of cardiac toxicity arising from cancer treatments. Moreover, they discussed how difficulties in managing strong and overwhelming affects, related to potential deficits in emotion regulation mechanisms may influence the connections between specific personality features and physical conditions. According to their perspective, it is necessary to adopt a multidisciplinary and personalized approach, based on an accurate and articulated psychological assessment that takes into account the patients' personality, mental functioning, and symptom patterns (cfr., Lingiardi and McWilliams, 2015; Hilsenroth et al., 2018).

Moving toward the empirical investigations of the present Special Issue, the work of Lanzara et al. explored the relationship between alexithymia and somatization in chronic pain (CP) patients. The authors showed that alexithymia, especially difficulty in identifying feelings (DIF), is highly associated with somatic manifestations, representing one of the major factors for somatization risk in CP patients. Moreover, testing a complex sequential mediation model, they suggested that DIF directly affects somatization and through the relevant contribution of emotional distress, pain interference, and mental health-related quality of life (HRQL) as mediating factors. The clinical implications of these results are addressed in more detail. Finally, the authors emphasized the importance of tailoring individualized interventions, focused on specific features of CP patients such as the difficulties in the cognitive processing of emotions and distress and tendency to somatization.

In their research, Buratta et al. explored the link between personality characteristics and overweight/obesity, a widespread health condition associated with numerous medical complications and mortality risks. The results showed that patients with overweight/obesity reported greater levels of somatic complaints, depression, and borderline features than the control group. Moreover, individuals with obesity seemed to establish more negative and unstable interpersonal relationships. The authors recommended the importance of assessing personality and interpersonal functioning in order to reduce the risk for negative health outcomes and worse prognosis.

Finally, Mokhtar et al.'s research concludes the Special Issue. The authors investigated depressive characteristics in patients with irritable bowel syndrome belonging to the subtype with constipation (IBS-C). The findings suggested that IBS-C is associated with a moderately high prevalence of depressive features. Given their impact on the quality of life and the risk of developing a major depressive episode, the authors suggested the need for providing a complex psychological assessment of patients with IBS-C that is very useful to identify individualized treatments.

The personalized medicine approach is progressively taking place as a new work method in clinical settings. We think that incorporating issues related to the personality characteristics in the treatments would help a multidisciplinary work with patients, probably improving the adherence to medical prescription and their satisfaction. Overall, all the contributions included in the present Special Issue suggest that a careful examination of multiple factors linking personality and diseases could be very useful to plan patient-tailored interventions, promote health outcomes, and enhance best practices in different medical contexts. In addition, future research will benefit from continuing the comprehensive exploration of how personality may contribute to individuals' mental and psychical well-being.

## Author Contributions

FG, AT, MP, and MB equally collaborated to the writing and revision of the editorial, with substantial, direct, and intellectual contribution to the work.

## Conflict of Interest

The authors declare that the research was conducted in the absence of any commercial or financial relationships that could be construed as a potential conflict of interest.

## Publisher's Note

All claims expressed in this article are solely those of the authors and do not necessarily represent those of their affiliated organizations, or those of the publisher, the editors and the reviewers. Any product that may be evaluated in this article, or claim that may be made by its manufacturer, is not guaranteed or endorsed by the publisher.
